# Effect of recombinant human platelet-derived growth factor-BB-coated sutures on Achilles tendon healing in a rat model: A histological and biomechanical study

**DOI:** 10.1177/2041731412453577

**Published:** 2012-07-02

**Authors:** Stephen H Cummings, Daniel A Grande, Christopher K Hee, Hans K Kestler, Colleen M Roden, Neil V Shah, Pasquale Razzano, David M Dines, Nadeen O Chahine, Joshua S Dines

**Affiliations:** 1 Department of Orthopaedic Surgery, Long Island Jewish Medical Center, New Hyde Park, NY, USA; 2 Laboratory of Orthopaedic Research, Feinstein Institute for Medical Research, North Shore Long Island Jewish Health System, Manhasset, NY, USA; 3 BioMimetic Therapeutics, Inc., Franklin, TN, USA; 4 Sports Medicine and Shoulder Service, Hospital for Special Surgery, New York, NY, USA; 5 Biomechanics & Bioengineering Research Laboratory, Feinstein Institute for Medical Research, North Shore Long Island Jewish Health System, Manhasset, NY, USA

**Keywords:** recombinant human platelet-derived growth factor-BB, suture, tendon repair, biomechanics, histology

## Abstract

**Purpose:**

Repairing tendon injuries with recombinant human platelet-derived growth factor-BB has potential for improving surgical outcomes. Augmentation of sutures, a critical component of surgical tendon repair, by coating with growth factors may provide a clinically useful therapeutic device for improving tendon repair. Therefore, the purpose of this study was to (a) coat Vicryl sutures with a defined dose of recombinant human platelet-derived growth factor-BB without additional coating excipients (e.g. gelatin), (b) quantify the recombinant human platelet-derived growth factor-BB released from the suture, and (c) use the recombinant human platelet-derived growth factor-BB-coated sutures to enhance tendon repair in a rat Achilles tendon transection model.

**Methods:**

Vicryl sutures were coated with 0, 0.3, 1.0, and 10.0 mg/mL concentrations of recombinant human platelet-derived growth factor-BB using a dip-coating process. In vitro release was quantified by an enzyme-linked immunosorbent assay. Acutely transected rat Achilles tendons were repaired using one of the four suture groups (*n* = 12 per group). Four weeks following repair, the tensile biomechanical and histological (i.e. collagen organization and angiogenesis) properties were determined.

**Results:**

A dose-dependent bolus release of recombinant human platelet-derived growth factor-BB occurred within the first hour in vitro, followed by a gradual release over 48 h. There was a significant increase in ultimate tensile strength (*p* < 0.01) in the two highest recombinant human platelet-derived growth factor-BB dose groups (1.9 ± 0.5 and 2.1 ± 0.5 MPa) relative to controls (1.0 ± 0.2 MPa). The modulus significantly increased (*p* = 0.031) with the highest recombinant human platelet-derived growth factor-BB dose group (7.2 ± 3.8 MPa) relative to all other groups (control: 3.5 ± 0.9 MPa). No significant differences were identified for the maximum load or stiffness. The histological collagen and angiogenesis scores were comparable in all groups, although there was a trend for improved collagen organization in the recombinant human platelet-derived growth factor-BB-treated groups (*p* = 0.054).

**Conclusions:**

The results of this study suggest that recombinant human platelet-derived growth factor-BB can be used to reproducibly coat Vicryl sutures and improve remodeling in a rat Achilles tendon transection model by significantly decreasing the resulting cross-sectional area, thus improving the material properties of the repaired tendon.

## Introduction

The Achilles tendon is the thickest and strongest tendon in the human body, which allows it to support high loads.^1^ However, the mechanical loading environment in which the Achilles tendon functions makes it prone to degenerative changes, including changes in fiber structure/arrangement, cellularity, vascularity, and collagen and glycosaminoglycan content, which can ultimately lead to rupture of the tendon.^2^ Treatment for Achilles tendon ruptures consists of either nonoperative or surgical interventions, with the decision often left to the preference of the surgeon. Surgical intervention results in lower rerupture rates compared to nonoperative treatment, but carries an increased risk of complications (e.g. sural nerve injury, infection, and delayed wound healing) associated with the procedure.^3–10^ Although the reported incidence of rerupture following surgical treatment is relatively low (1.7%–5.4%),^1,3–10^ many patients still experience functional deficits, as evidenced by the percentage of patients who report “good or excellent” outcomes (84%)^6^ or who were able to return to their previous level of activity (71%–73%).^3,7^ As the ultimate goal of Achilles tendon repair is to return the patient to prerupture levels of activity, there is a compelling need to improve contemporary Achilles tendon repair, especially for active patients who seek a rapid return to function.^1^ Augmentation of the biological repair process with recombinant growth factors is an exciting option for improving tendon healing, accelerating return to activity, improving clinical outcomes, and decreasing posttreatment morbidity.

Biological augmentation of tendon healing with growth factors has shown promising preclinical results.^11^ One such growth factor, platelet-derived growth factor-BB (PDGF-BB) homodimer, is endogenously expressed in tendon following injury.^12–14^ PDGF-BB promotes chemotaxis and mitogenesis of mesenchymal cells, including tendon fibroblasts and mesenchymal stem cells,^15–19^ which deposit and remodel the extracellular matrix and improve biomechanical properties of the repaired tendon. Recombinant human platelet-derived growth factor-BB (rhPDGF-BB) has successfully promoted tendon healing, including improving the mechanical function, collagen organization, and vascularity.^18,20–25^ While rhPDGF-BB has been previously shown to be effective for tendon healing, optimization of the method of delivery is expected to further improve the treatment of tendon ruptures by matching the application of therapeutic biologicals and the wound healing process. A vehicle for rhPDGF-BB delivery therefore must fulfill tight wound healing parameters, which includes delivering the molecule to the site of repair at the correct time and in optimum doses for therapeutic efficacy. Moreover, a surgically convenient delivery system for the rhPDGF-BB would improve clinical utility. Consequently, we utilized coated sutures as the mode of delivery for rhPDGF-BB.

Sutures are almost universally used in surgical interventions to repair torn tendons, as they provide initial mechanical stability to the repair, making them an ideal delivery vehicle. Growth factor-coated Vicryl sutures have been used to improve healing in rat Achilles tendon^26^ and sheep anterior cruciate ligament (ACL) repair models.^27^ A single dose of growth factor was used in each study and the in vivo dose of growth factor was inferred based on the concentration of the coating solution, but the actual amount coated on the suture was not quantified. Consequently, the delivered dose was not determined. The actual delivered dose is crucial for predicting outcomes and translating doses from animal studies to human trials.

Dines et al.^28^ used growth/differentiation factor-5 (GDF-5)–coated Vicryl sutures, quantified for growth factor loading, to improve the outcomes of Achilles tendon repair in rats. Similarly, Uggen et al.^24^ demonstrated that treatment with FiberWire sutures coated with quantified amounts of rhPDGF-BB resulted in improved healing in an ovine model of rotator cuff repair. In both studies, 10% gelatin was added to the coating solution to improve the efficiency of the growth factor coating. While addition of gelatin to the coated suture is not necessarily deleterious to repair, it can present regulatory (i.e. safety and toxicity testing) and manufacturing complications. Although it may be advantageous to use sutures coated with only the growth factor solution, the efficacy of this approach has not been previously explored for Achilles repair.

The purpose of the current study was to (a) coat Vicryl sutures with a defined dose of rhPDGF-BB without additional coating excipients (e.g. gelatin), (b) quantify the amount of rhPDGF-BB released from the suture, and (c) use the rhPDGF-BB-coated sutures to enhance tendon repair in a rat Achilles tendon transection model. We hypothesized that (1) Vicryl sutures could be successfully coated with reproducible amounts of rhPDGF-BB by varying the initial dip-coating solution concentration and (2) rhPDGF-BB-coated sutures would enhance rat Achilles tendon repair in a dose-dependent manner.

## Materials and methods

The study was carried out in two parts: (a) in vitro suture coating and quantification and (b) in vivo Achilles tendon repair in a rat model. The in vivo study was Institutional Animal Care and Use Committee (IACUC) approved.

### Part 1: suture coating

Four groups of 4-0 Vicryl sutures (Ethicon, Somerville, NJ) were coated with 20 mM sodium acetate (Group 1, vehicle control), 0.3 mg/mL rhPDGF-BB in sodium acetate (Group 2), 1.0 mg/mL rhPDGF-BB in sodium acetate (Group 3), or 10.0 mg/mL rhPDGF-BB in sodium acetate (Group 4), using a dip-coating process as described previously.^28,29^ Sodium acetate is an inert solution that allows rhPDGF-BB to be in solution during the suture coating process. Sodium acetate does not interact with the suture, rhPDGF-BB, or the local tissue. Briefly, after treatment with 70% ethanol, the sutures were submerged in sodium acetate, with or without rhPDGF-BB (0, 0.3, 1.0, and 10.0 mg/mL), for 30 min and then air-dried. Unlike the process described by Dines et al.,^28^ no gelatin was used in the coating solution. Sutures were trimmed to 15 cm lengths for the in vivo study with the remainder used for in vitro analysis.

### In vitro rhPDGF-BB release

To determine the amount of rhPDGF-BB released from the suture, an in vitro elution assay was performed as described previously.^24^ Coated sutures (*n* = 5 per group) were placed in 1 mL of elution buffer (minimum essential medium with 2% fetal bovine serum, 1% penicillin–streptomycin, 1% L-glutamine, and 1% 4-(2-hydroxyethyl)-1-piperazineethanesulfonic acid (HEPES) buffer) and incubated at 37°C on a rocking platform. The buffer was fully exchanged at 1, 6, 24, and 48 h. The total rhPDGF-BB released at each time point was determined using a human PDGF-BB DuoSet enzyme-linked immunosorbent assay (ELISA) according to manufacturer’s instructions (R&D Systems, Minneapolis, MN). Relative bioactivity was evaluated in an in vitro proliferation assay comparing the dose–response curves of MG-63 osteosarcoma cells. Serial dilutions of the rhPDGF-BB eluted from the sutures or the original rhPDGF-BB coating solution (dose range: 2–600 ng/mL) was added to MG-63 cells plated in serum-free medium. Cells were incubated for 3 days and the relative bioactivity of the samples was calculated by comparing the slopes of the dose–response curves using the original rhPDGF-BB coating solution as the reference.

### Part II: surgical procedure

Forty-eight Sprague–Dawley rats (350–400 g) were randomized to one of the four treatment groups (*n* = 12 per group) as described above. All surgeries were performed under sterile conditions. Following blunt dissection to expose the Achilles tendon, the tendon was transected proximal to its insertion on the calcaneus and immediately repaired using one modified Mason–Allen stitch and one simple interrupted stitch using sutures from one of the four treatment groups. The remaining, unused suture was measured to determine the length of coated suture implanted. The skin was closed with interrupted uncoated Vicryl sutures and the animals were allowed to ambulate normally. After 4 weeks, the rats were killed and the tendons, including a bone block from the calcaneus and the proximal gastroc-soleus muscle complex, were harvested. The specimens were randomly assigned to biomechanical (*n* = 8 per group, fresh frozen) or histological analysis (*n* = 4 per group, formalin fixed).

### Biomechanics

Uniaxial tensile biomechanical analysis was performed using an Instron system (Model No. 5566) with a 100 N load cell (accuracy: ±0.5%).^30^ Samples were thawed at 4°C in phosphate-buffered saline (PBS) containing protease inhibitors, dissected to remove excess muscle, and the calcaneus and gastroc-soleus ends secured between pneumatic grips in a PBS bath. Samples were preloaded (1 N) in tension and sample dimensions at the midpoint of the gage length were measured using calipers. Cross-sectional area (CSA) was calculated based on rectangular geometry to allow for comparative analysis across the samples from each group.^28,30^ Specimens were pulled to failure at a strain rate of 0.25% per second in precise displacement control and the resulting load–extension data were collected at 10 Hz. The linear stiffness and elastic modulus were determined from the linear portion of the load–displacement or stress–strain curve, respectively, using a best-fit analysis. The maximum load and ultimate tensile strength were also determined. Biomechanical data are presented as mean ± standard deviation (SD).

### Histology

Tendon specimens were detached from the calcaneus at the Achilles tendon insertion site and the repaired Achilles tendons were processed and embedded in paraffin. Parasagittal sections were stained with either Mallory’s trichrome or Sirius red. Slides were imaged and scored by three observers, masked to treatment, using a scoring system for collagen organization and degree of angiogenesis (Table 1), as described previously.^28,31^ Three sections were scored and the average scores from the three observers were taken. Histology scores are presented as median (range).

**Table 1.** Histology scoring system^28^

Score	Collagen organization	Angiogenesis
1	Normal collagen oriented tangentially	Normal tendon tissue
2	Mild changes with collagen fibers, less than 25% disorganized	Increased presence of capillaries
3	Moderate changes with collagen fibers, less than 50% disorganized	Moderate infiltration of tissue with vessels
4	Marked changes in collagen, more than 50% disorganized	–

### Statistical analysis

The amount of rhPDGF-BB released in vitro was analyzed using a repeated measures analysis of variance (ANOVA), with time as the repeated measure. The cumulative amount of rhPDGF-BB released and the total dose delivered versus the initial coating concentration were fit with a nonlinear (log–log scale) least squares fit. A one-way ANOVA with a Holm–Sidak multiple comparison test was used to determine differences among groups for the biomechanical parameters. Statistical analysis on the histology grading scores was performed using an ANOVA on the ranks. Significance was set at *p* ≤ 0.05.

## Results

### In vitro release of rhPDGF-BB

**Figure 1.**
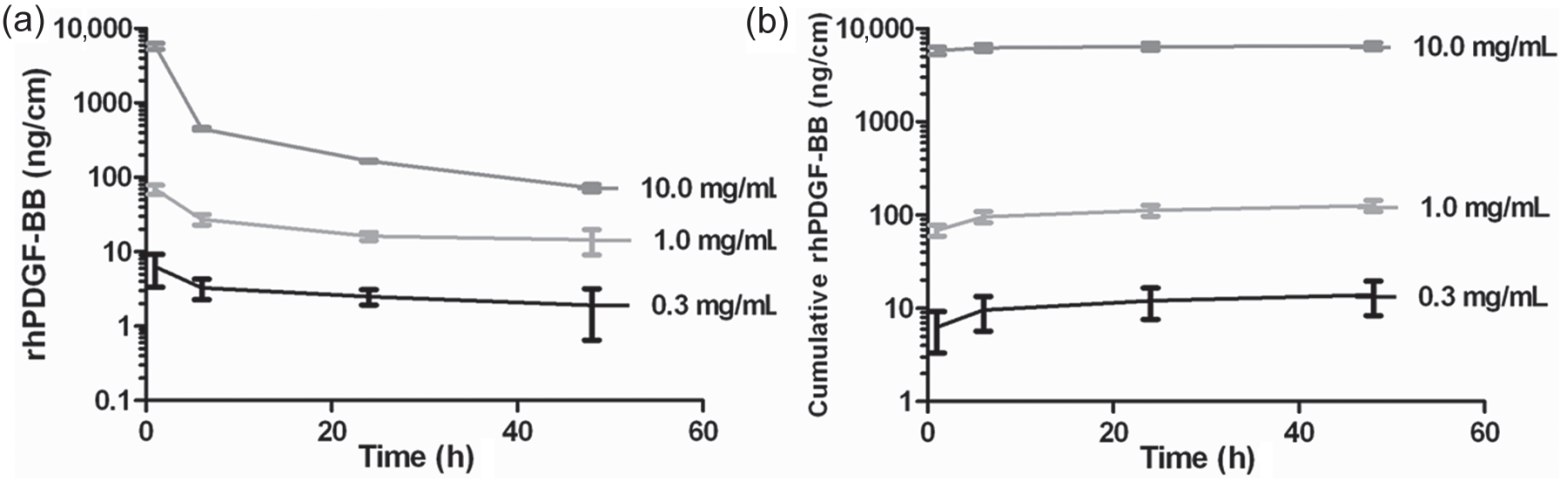
(a) Amount of rhPDGF-BB released from the coated sutures at each time point in vitro and (b) the cumulative amount of rhPDGF-BB released from the coated sutures in vitro plotted on a semilogarithmic scale. The amounts released in vitro were normalized by the length of the suture (ng/cm). rhPDGF-BB: recombinant human platelet-derived growth factor-BB.

The amount of rhPDGF-BB released at each time point was normalized by the length of the suture (ng/cm) (Figure 1). The amount of rhPDGF-BB detected in the elution buffer of Group 1 (vehicle control) sutures was at the lower limit of detection for the assay. A dose-dependent bolus release of rhPDGF-BB from the sutures was observed after the first hour of incubation (Figure 1(a)). The initial bolus was followed by a continuous, gradual release of rhPDGF-BB through the 48-h time point. The cumulative amount of rhPDGF-BB released over the 48-h incubation was dose-dependent, with more rhPDGF-BB released with higher initial dip-coating concentrations (Figure 1(b)). Group 4 was significantly increased (*p* < 0.001) relative to Groups 2 and 3. Groups 2 and 3 were also significantly different from each other (*p* < 0.05). The cumulative amount of rhPDGF-BB released (*Y*, ng/cm) was proportional (*r*^2^ = 0.9575) to the initial coating concentration (*X*, mg/mL) by the following equation: *Y* = 126.47 × *X*^1.711^. The rhPDGF-BB released from the sutures coated with 10 mg/mL retained 71.8% ± 3.6% of its bioactivity compared to the rhPDGF-BB coating solution, as measured in an in vitro proliferation assay. The concentrations of rhPDGF-BB released from the 0.3 and 1.0 mg/mL groups were too low to be tested in this bioactivity assay.

### Surgical and observations

All animals responded well to the surgery, with the exception of one animal in Group 3 that died overnight following surgery secondary to complications from anesthesia. The length of suture used for the repair was consistent among groups (Group 1: 4.8 ± 0.5 cm, Group 2: 4.7 ± 0.7 cm, Group 3: 4.8 ± 0.3 cm, and Group 4: 4.8 ± 0.5 cm; *p* = 0.94). Based on the cumulative amount of rhPDGF-BB released in vitro and the lengths of suture, the in vivo doses for each group were calculated to be as follows: Group 1: 0 ng, Group 2: 66.0 ± 61.2 ng, Group 3: 602.5 ± 190.9 ng, and Group 4: 31,342.6 ± 6774.5 ng. Similar to the cumulative amount of rhPDGF-BB released, the in vivo dose delivered (*Y*, ng) was proportional (*r*^2^ = 0.9859) to the initial coating concentration (*X*, mg/mL) by the following equation: *Y* = 571.48 × *X*^1.732^.

### Biomechanics

The specimens used for biomechanical analysis were distributed as follows: *n* = 7 (Groups 1–3) and *n* = 6 (Group 4). No significant differences in the structural properties of the repaired tendons (maximum load and stiffness) were observed (Table 2). However, the mean values for the ultimate load (*p* = 0.16) and stiffness (*p* = 0.21) were consistently higher in the groups receiving rhPDGF-BB (Groups 2–4), relative to the control group (Group 1). A significant decrease (*p* < 0.01) in the CSA was observed in Group 4 compared to Groups 1 and 2 (Table 2). The CSA was also significantly decreased (*p* < 0.01) in Group 3 relative to Group 2. The decrease in CSA resulted in significant differences in the material properties of the repaired tendons (i.e. ultimate strength and elastic modulus) (Table 2). There was a significant increase (*p* < 0.001) in the ultimate tensile strength in Group 4 relative to Groups 1 and 2 and in Group 3 (1.9 ± 0.5 MPa) compared to Group 1. The elastic modulus was significantly increased (*p* = 0.031) in Group 4 compared to all the other groups. Groups 1–3 were not statistically different from each other.

**Table 2.** Tensile biomechanical properties of repaired Achilles tendons

Group	Maximum load (N)	Stiffness (N/mm)	Ultimate strength (MPa)	Modulus (MPa)	CSA (cm^2^)
1	22.1 ± 4.8	6.7 ± 1.0	1.0 ± 0.2	3.5 ± 0.9	0.21 ± 0.03
2	31.6 ± 9.3	8.9 ± 2.1	1.4 ± 0.3	4.4 ± 1.0	0.23 ± 0.04
3	28.0 ± 9.8	8.1 ± 2.5	1.9 ± 0.5^a^	4.9 ± 1.9	0.17 ± 0.02^b^
4	27.5 ± 4.3	7.9 ± 1.7	2.1 ± 0.5^c^	7.2 ± 3.8^d^	0.14 ± 0.05^c^

a *p* ≤ 0.001 compared to Group 1.

b *p* ≤ 0.01 compared to Group 2.

c *p* ≤ 0.01 compared to Groups 1 and 2.

d *p* ≤ 0.001 compared to all other groups.

CSA: cross-sectional area.

### Histology

**Figure 2.**
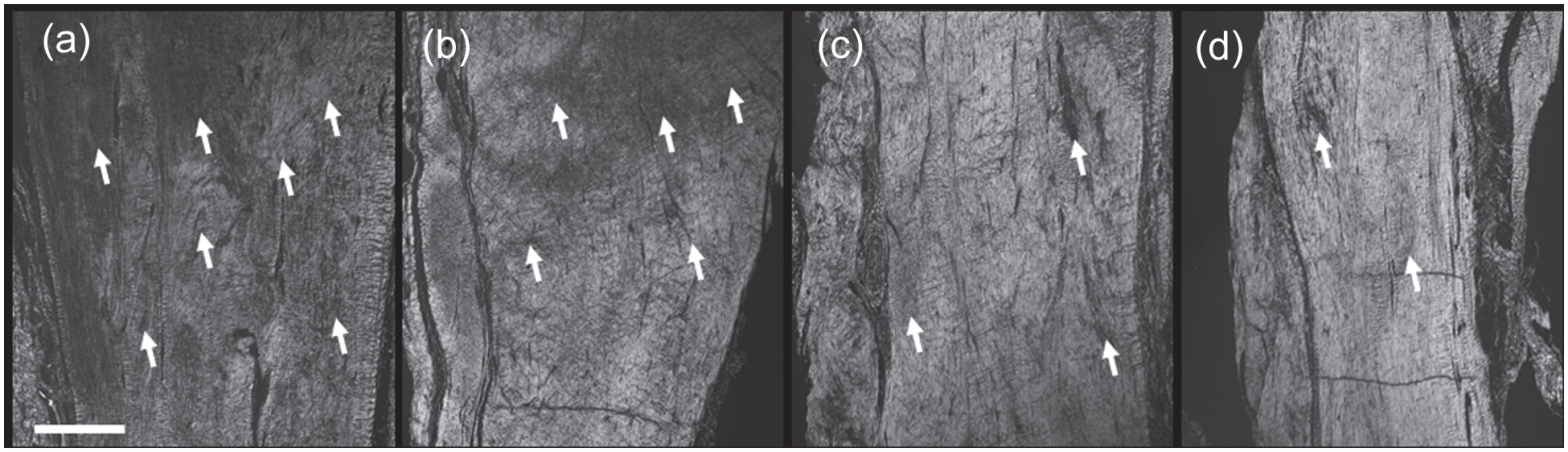
Representative polarized light microscopy images showing collagen organization in the repaired regions of the tendons for (a) Group 1, (b) Group 2, (c) Group 3, and (d) Group 4. Bright exposure under polarized light indicates collagen that is highly aligned along the direction of loading, while areas of darker exposure (arrows) indicate regions of decreased collagen organization/alignment. (b–d) A trend for increased collagen organization (decreased observations of unorganized tissue) was observed in the rhPDGF-BB-treated groups (scale bar = 500 µm). rhPDGF-BB: recombinant human platelet-derived growth factor-BB.

Histology was performed on four animals from each group (except Group 3, in which *n* = 3). There was a trend for improved collagen organization in the rhPDGF-BB-treated groups (*p* = 0.054), with lower median scores (Table 3 and Figure 2) in the tendons in which rhPDGF-BB-coated sutures were used. Lower median scores were associated with more aligned collagen fibers. The median angiogenesis score was increased in the highest rhPDGF-BB dose group (Table 3), suggesting increased vascularity; however, this increase did not reach significance (*p* = 0.123).

**Table 3.** Histology scores

Group	Collagen organization	Angiogenesis
1	1.6 (1.2–2.0)	1.3 (1.0–1.5)
2	1.0 (0.5–1.7)	1.3 (1.0–1.8)
3	0.8 (0.7–0.8)	1.0 (0.8–1.3)
4	1.0 (0.8–1.5)	1.7 (1.3–1.8)

## Discussion

The purpose of the current study was to examine the release kinetics of rhPDGF-BB-coated Vicryl sutures and the effect of these coated sutures on tendon repair in a rat Achilles tendon transection model. Our findings indicate that rhPDGF-BB can be successfully coated on Vicryl sutures to specific dosing tolerances without the use of excipients, such as gelatin. In addition, dose-dependent decrease in the CSA and increase in the material properties of Achilles tendons repaired with rhPDGF-BB-coated sutures suggest that rhPDGF-BB may be effective at modulating the healing response of torn Achilles tendons.

Quantification of rhPDGF-BB coated on the sutures is of critical importance in order to determine the efficacious dose for tendon healing and thus produce a precise therapeutic effect. Our findings indicate that the amount of rhPDGF-BB coated on the sutures was proportional to the initial coating concentration, allowing consistent doses to be delivered in vivo. Increasing initial rhPDGF-BB concentrations resulted in more rhPDGF-BB being coated on the suture material over the range of initial concentrations used in this study (0.3, 1.0, and 10.0 mg/mL). While a theoretical saturation limit would be expected, 10 mg/mL is the maximum feasible concentration of rhPDGF-BB in solution, so the initial concentration required to reach this saturation limit is unknown. An initial bolus release of the rhPDGF-BB was observed followed by a continuous, gradual release of rhPDGF-BB over a 48-h window, which is consistent with the release of rhPDGF-BB from FiberWire sutures coated with gelatin as reported previously by Uggen et al.^24^ Moreover, the rhPDGF-BB released from the sutures retained approximately 70% of its bioactivity during this time frame. While the in vitro release was used to estimate the in vivo dose, the actual amount and time course of release were not determined in vivo. The half-life of rhPDGF-BB in vivo has been shown to be less than 1 h in the systemic circulation, suggesting that there would not be a systemic effect of locally delivered rhPDGF-BB. Even with the fast systemic clearance, the local tissue concentrations of rhPDGF-BB likely remain elevated for a longer period of time due to the regulation of PDGF-BB clearance by *α*_2_-macroglobulin and the binding of PDGF-BB to extracellular matrix proteins.^32–34^ An elevated local concentration would influence the proliferative phase of the tendon-healing cascade by increasing migration and proliferation (both mechanisms of action of PDGF-BB) of tenocytes and progenitor cells, resulting in an increased number of cells available to impact the later phases of tendon repair and remodeling. Further characterization of the in vivo pharmacokinetic release profile will help to elucidate, in an even more precise manner, the in vivo parameters required for optimum rhPDGF-BB efficacy. Moreover, the outcome data further suggest that rhPDGF-BB can be coated reproducibly on Vicryl sutures without additional coating excipients (e.g. gelatin) in doses necessary to improve tendon healing.

The primary goal of repairing ruptured tendons is to restore the structure and mechanical function of the injured tissue. The structural properties (maximum load and stiffness), which indicate the overall ability of the tendon to support a load, were increased, on average, following rhPDGF-BB treatment; however, the increase did not reach statistical significance (*p* = 0.16 and 0.21). Significantly increased material properties (ultimate strength and elastic modulus), which are a measure of the quality of the tissue, were observed with the highest dose of rhPDGF-BB. The dose-dependent increase in material properties but not structural properties is likely to be a result of the significant decrease in CSA. Consequently, improvements in the material properties are indicative of improved remodeling and reorganization of tendon repaired with highest dose of rhPDGF-BB. While the use of noncontact measures of sample dimensions may potentially improve measurement accuracy, the use of contact method (calipers) have been used previously in this model^28,30^ and did not interfere with the detection of statistical significance in the CSA at the midsegment of the specimens.

Our results indicate that the histological collagen and angiogenesis scores were comparable in all groups, although there was a trend for improved collagen organization in the rhPDGF-BB-treated groups (*p* = 0.054). Previous studies have indicated that a sample size of four animals was sufficient to observe significant differences in the histological outcomes measured here.^28,31^ Additional measures, such as fibroblast density, collagen cross-linking, or collagen fibril diameter, may also impact the mechanical properties; however, those measurements were beyond the scope of this study. Although the histological scores did not reach statistical significance in this study, the overall trend of the histological outcomes, combined with the decrease in CSA and increase in material properties, suggests that the highest dose of rhPDGF-BB promoted improved remodeling relative to the tendons repaired with control suture. As such, these results provide a compelling indication that rhPDGF-BB improves the remodeling of torn Achilles tendons when the growth factor is delivered locally coated on sutures.

Previous studies have shown that rhPDGF-BB can successfully promote tendon healing, including improving the mechanical function, collagen organization, and vascularity.^18,20–25^ In addition to investigating different injury models, including tendon-to-bone (i.e. sheep rotator cuff) and tendon-to-tendon (i.e. canine flexor tendons), these previous studies have looked at a variety of delivery systems and dosages. However, despite the differences in the injury models, dosages, and delivery systems, the results of the current study are consistent with those found previously. For instance, in a series of studies that used a fibrin matrix to deliver rhPDGF-BB to a flexor tendon,^18,21,22^ the authors noted improvements in the functional properties (e.g. joint rotation and excursion) and the histological appearance (e.g. fibroblast density, type I collagen, and collagen cross-linking). Although the functional properties of the tendon were improved, the tensile properties were not increased in these prior studies. This may be due to the mechanical function of the specific tendons being investigated or differences in the dosage of rhPDGF-BB (100 ng) used as compared to other studies investigating tendon healing. Including the current study, the efficacious dose that improves the tensile properties has ranged from 1000 ng in a rat patellar tendon defect^20^ to 12–150 µg in a sheep rotator cuff model.^24,25^ The differences in magnitude between the rat and the sheep may also be due to the size of the animal being studied. Conversely, a high dose of rhPDGF-BB (500 µg) was shown to be less efficacious at improving the mechanical properties than lower doses (75 and 150 µg) and no different than control repairs in the sheep rotator cuff model.^25^ Such a biphasic phenomenon was not observed in this study, reinforcing that the doses delivered using the coated sutures were sufficient for improving tendon repair without applying too high of a dose. Another result of using rhPDGF-BB in tendon repair models is improvement of the morphological properties of the tendon, such as collagen content, collagen alignment, collagen cross-linking, and vascularity. Previous studies consistently demonstrated improvements in these areas,^18,20–25^ which is consistent with the overall trend of the histological outcomes demonstrated in this study. Taken together, the results of this study demonstrate that coated sutures are a practical method of delivering therapeutic doses of rhPDGF-BB in tendon repair applications.

The rat model for studying Achilles tendon repair is well established in the orthopedic literature.^26,28,30,31,35,36^ As with any animal model, there are some limitations in this study that preclude direct extrapolation of rhPDGF-BB-coated sutures directly to use in the clinical setting. In smaller animals, the mechanical loading requirements are not as rigorous as they are in humans, which is reflected in the size and type of suture that was used. In this study, a 4-0 degradable Vicryl suture was coated with rhPDGF-BB which does not match the clinical use of nondegradable reinforced sutures that provide mechanical stability. Preliminary studies demonstrated that higher doses of rhPDGF-BB could be coated onto degradable sutures as compared to nondegradable sutures when no additional excipients were used. Gelatin was required to coat appreciable amounts of rhPDGF-BB on #2 FiberWire sutures (1730.8 ± 338.2 ng/cm with gelatin vs 56.8 ± 14.4 ng/cm without gelatin), whereas sufficient amounts of rhPDGF-BB could be coated on 4-0 Vicryl sutures (1100.1 ± 57.0 ng/cm with gelatin vs 703.2 ± 138.65 ng/cm without gelatin). While the sutures used here are not typical for Achilles tendon repair in the clinic, this study does underscore the fact that delivery of rhPDGF-BB is possible using coated sutures and is able to augment the biological repair of the rat Achilles tendon, even without the additional support that would be provided by nondegradable sutures. In addition to differences in mechanical loading requirements, the ability to control the post-repair loading is not consistent with the human condition, where different regimens of casting/bracing and physical therapy can be prescribed. The inability to control post-repair loading in this model creates a challenging environment for repair that would be analogous to a noncompliant patient, which can present clinical challenges that are difficult to overcome with standard repair. Furthermore, there are differences between the acute laceration model used in this study and the clinical presentation of tendon ruptures. Namely, the degenerative process that precedes rupture in the clinic^2^ is not observed in this model. While repair of an acutely lacerated tendon may not be indicative of the degenerated condition, this model is still useful for evaluating the role of growth factors in tendon healing and repair. One limitation in this study is that a time course of healing was not investigated. A single time point of 4 weeks was chosen to evaluate the effect of rhPDGF-BB on tendon properties, as 4 weeks has been used previously in the rat Achilles tendon transection model to demonstrate the efficacy of growth factors or cell therapies on the biomechanical properties of the repaired tendons.^26,30,35^ While additional time points will help assess the rate of healing using this model, the current study was used to identify a specific dose that is capable of modulating the healing response of an Achilles transection. Future studies will examine the effect of rhPDGF-BB on the rate of repair.

In conclusion, to our knowledge, this is the first study to report on rhPDGF-BB-coated sutures as a therapy for improving the material properties of healing rat Achilles tendon in a dose-dependent manner. Continued progression with rhPDGF-BB-coated sutures in animal studies, such as in the ovine rotator cuff repair model evaluated by Uggen et al.^24^, may be an antecedent step to human applications for improving, and potentially accelerating, healing following tendon injury. The results of this study suggest that rhPDGF-BB-coated sutures improve tendon remodeling and increase function at 4 weeks following tendon repair in a rat Achilles tendon model. Recombinant human growth factors, such as rhPDGF-BB, have made a beneficial impact in clinical care^37–39^ and new methods for their delivery are likely to grow with the ever increasing supply of biomaterials for improved wound repair and delivery.

## References

[1] Maffulli N Rupture of the Achilles tendon. J Bone Joint Surg Am 1999; 81(7): 1019–1036 10428136 10.2106/00004623-199907000-00017

[2] Mafulli N Barrass V Ewen SWB Light microscopic histology of Achilles tendon ruptures: a comparison with unruptured tendons. Am J Sports Med 2000; 28(6): 857–863 11101109 10.1177/03635465000280061401

[3] Lo IK Kirkley A Nonweiler B Operative versus nonoperative treatment of acute Achilles tendon ruptures: a quantitative review. Clin J Sport Med 1997; 7: 207–211 9262889 10.1097/00042752-199707000-00010

[4] Khan RJK Fick D Keogh A Treatment of acute Achilles tendon ruptures: a meta-analysis of randomized, controlled trials. J Bone Joint Surg Am 2005; 87(10): 2202–2210 16203884 10.2106/JBJS.D.03049

[5] Lynch RM. Achilles tendon rupture: surgical versus non-surgical treatment. Accid Emerg Nurs 2004; 12: 149–158 15234712 10.1016/j.aaen.2003.11.004

[6] McMahon SE Smith TO Hing CB A meta-analysis of randomised controlled trials comparing conventional to minimally invasive approaches for repair of an Achilles tendon rupture. Foot Ankle Surg 2011; 17: 211–217 22017889 10.1016/j.fas.2010.11.001

[7] Bhandari M Guyatt GH Siddiqui F Treatment of acute Achilles tendon ruptures: a systematic overview and metaanalysis. Clin Orthop Rel Res 2002; 400: 190–200 10.1097/00003086-200207000-00024 12072762

[8] Inglis A Scott W Sculco T Ruptures of the tendo achillis. An objective assessment of surgical and non-surgical treatment. J Bone Joint Surg Am 1976; 58(7): 990–993 977631

[9] Nistor L. Surgical and nonsurgical treatment of Achilles tendon rupture: a prospective randomized trial. J Bone Joint Surg Am 1981; 63(3): 394–399 7204438

[10] Willits K Amendola A Bryant D Operative versus nonoperative treatment of acute Achilles tendon ruptures: a multicenter randomized trial using accelerated functional rehabilitation. J Bone Joint Surg Am 2010; 92(17): 2767–2775 21037028 10.2106/JBJS.I.01401

[11] Longo UG Lamberti A Maffulli N Tissue engineered biological augmentation for tendon healing: a systematic review. Br Med Bull 2011; 98: 31–59 20851817 10.1093/bmb/ldq030

[12] Duffy FJ Seiler JG Gelberman RH Growth factors and canine flexor tendon healing: initial studies in uninjured and repair models. J Hand Surg Am 1995; 20: 645–649 7594295 10.1016/S0363-5023(05)80284-9

[13] Tsubone T Moran S Amadio PC Expression of growth factors in canine flexor tendon after laceration in vivo. Ann Plast Surg 2004; 53(4): 393–397 15385778 10.1097/01.sap.0000125501.72773.01

[14] Wurgler-Hauri CC Dourte LM Baradet TC Temporal expression of 8 growth factors in tendon-to-bone healing in a rat supraspinatus model. J Shoulder Elbow Surg 2007; 16(5): S198–S203 17903711 10.1016/j.jse.2007.04.003 PMC4001791

[15] Hollinger JO Hart CE Hirsch SN Recombinant human platelet-derived growth factor: biology and clinical applications. J Bone Joint Surg Am 2008; 90: 48–54 18292357 10.2106/JBJS.G.01231

[16] Costa MA Wu C Pham BV Tissue engineering of flexor tendons: optimization of tenocyte proliferation using growth factor supplementation. Tissue Eng 2006; 12(7): 1937–1943 16889523 10.1089/ten.2006.12.1937

[17] Thomopoulos S Harwood F Silva M Effect of several growth factors on canine flexor tendon fibroblast proliferation and collagen synthesis in vitro. J Hand Surg Am 2005; 30(3): 441–447 15925149 10.1016/j.jhsa.2004.12.006

[18] Thomopoulos S Zaegel M Das R PDGF-BB released in tendon repair using a novel delivery system promotes cell proliferation and collagen remodeling. J Orthop Res 2007; 25(10): 1358–1368 17551975 10.1002/jor.20444

[19] Sakiyama-Elbert SE Das R Gelberman RH Controlled-release kinetics and biologic activity of platelet-derived growth factor-BB for use in flexor tendon repair. J Hand Surg Am 2008; 33(9): 1548–1557 18984337 10.1016/j.jhsa.2008.05.030 PMC2586996

[20] Chan BP Fu SC Qin L Supplementation-time dependence of growth factors in promoting tendon healing. Clin Orthop Relat Res 2006; 448:240–247 16826122 10.1097/01.blo.0000205875.97468.e4

[21] Thomopoulos S Das R Silva MJ Enhanced flexor tendon healing through controlled delivery of PDGF-BB. J Orthop Res 2009; 27(9): 1209–1215 19322789 10.1002/jor.20875 PMC2916020

[22] Gelberman R Thomopoulos S Sakiyama-Elbert S The early effects of sustained platelet-derived growth factor administration on the functional and structural properties of repaired intrasynovial flexor tendons: an in vivo biomechanic study at 3 weeks in canines. J Hand Surg Am 2007; 32(3): 373–379 17336846 10.1016/j.jhsa.2006.12.009

[23] Uggen JC Dines J Uggen CW Tendon gene therapy modulates the local repair environment in the shoulder. J Am Osteopath Assoc 2005; 105(1): 20–21 15710662

[24] Uggen C Dines J McGarry M The effect of recombinant human platelet-derived growth factor BB-coated sutures on rotator cuff healing in a sheep model. Arthroscopy 2010; 26(11): 1456–1462 20729027 10.1016/j.arthro.2010.02.025

[25] Hee CK Dines JS Dines DM Augmentation of a rotator cuff suture repair using rhPDGF-BB and a type I bovine collagen matrix in an ovine model. Am J Sports Med 2011; 39(8): 1630–1639 21555508 10.1177/0363546511404942

[26] Rickert M Jung M Adiyaman M Growth and differentiation factor 5 coated suture stimulates tendon healing in an Achilles tendon model in rats. Growth Factors 2001; 19: 115–126 11769971 10.3109/08977190109001080

[27] Weiler A Forster C Hunt P The influence of locally applied platelet-derived growth factor-BB on free tendon graft remodeling after anterior cruciate ligament reconstruction. Am J Sports Med 2004; 32(4): 881–891 15150033 10.1177/0363546503261711

[28] Dines JS Weber L Razzano P The effect of growth differentiation factor-5-coated sutures on tendon repair in a rat model. J Shoulder Elbow Surg 2007; 16(5 suppl): S215–S221 17507245 10.1016/j.jse.2007.03.001

[29] Dines JS Cross MB Dines D In vitro analysis of an rhGDF-5 suture coating process and the effects of rhGDF-5 on rat tendon fibroblasts. Growth Factors 2011; 29(1): 1–7 20969542 10.3109/08977194.2010.526605

[30] Daher RJ Chahine NO Razzano P Tendon repair augmented with a novel circulating stem cell population. Int J Clin Exp Med 2011; 4(3): 214–219 21977235 PMC3182514

[31] Rosenbaum AJ Wicker JF Dines JS Histologic stages of healing correlate with restoration of tensile strength in a model of experimental tendon repair. HSS J 2010; 6: 164–170 21886531 10.1007/s11420-009-9152-5 PMC2926361

[32] Solchaga LA Hee CK Roach S Safety of recombinant human platelet-derived growth factor-BB in Augment Bone Graft. J Tissue Eng 2012; 3(1): 2041731412442668 22511993 10.1177/2041731412442668 PMC3324841

[33] Martino MM Hubbell JA The 12th-14th type III repeats of fibronectin function as a highly promiscuous growth factor-binding domain. FASEB J 2010; 24(12): 4711–4721 20671107 10.1096/fj.09-151282

[34] Göhring W Sasaki T Heldin CH Mapping of the binding of platelet-derived growth factor to distinct domains of the basement membrane proteins BM-40 and perlecan and distinction from the BM-40 collagen-binding epitope. Eur J Biochem 1998; 255(1): 60–66 9692901 10.1046/j.1432-1327.1998.2550060.x

[35] Aspenberg P Virchenko O Platelet concentrate injection improves Achilles tendon repair in rats. Acta Orthop Scand 2004; 75(1): 93–99 15022816 10.1080/00016470410001708190

[36] Virchenko O Aspenberg P How can one platelet injection after tendon injury lead to a stronger tendon after 4 weeks? Acta Orthop 2006; 77(5): 806–812 17068715 10.1080/17453670610013033

[37] Nevins M Giannobile WV McGuire MK Platelet-derived growth factor stimulates bone fill and rate of attachment level gain: results of a large multicenter randomized controlled trial. J Periodontol 2005; 76: 2205–2215 16332231 10.1902/jop.2005.76.12.2205

[38] Kaigler D Avila G Wisner-Lynch L Platelet-derived growth factor applications in periodontal and peri-implant bone regeneration. Expert Opin Biol Ther 2011; 11(3): 1–11 21288185 10.1517/14712598.2011.554814 PMC3056074

[39] Daniels T DiGiovanni C Lau J Prospective clinical pilot trial in a single cohort group of rhPDGF in foot arthrodeses. Foot Ankle Int 2010; 31(6): 473–479 20557811 10.3113/FAI.2010.0473

